# Supplementary material for: Assessment of patient dose and optimization levels in chest and abdomen CR examinations at referral hospitals in Tanzania
Source: J Appl Clin Med Phys. 2015 Sep 8;16(5):435–41. doi: 10.1120/jacmp.v16i5.5614 (PMC5690155; doi:10.1120/jacmp.v16i5.5614)
Supplement: Supplementary file 1 — Supplementary Material [file ACM2-16-435-s001.doc]

**Abstract: The aim of this study was to evaluate the level of patient doses in chest and abdomen CR examinations, and that of optimisation at five facilities. The international code of practice for dosimetry in diagnostic radiology was applied to determine the entrance surface air kerma (ESAK) to patients. The level of optimisation was assessed from low contrast objects scores of phantom images at different exposures. The results show that mean ESAK varied from 0.16 to 0.37 mGy for chest PA and from 2 to 6 mGy for abdomen AP. Assuming similar patient and phantom attenuations, the optimisation performed at four facilities was consistent with phantom evaluations in terms of tube potential settings in use. However, all facilities seemed to operate at higher tube load values, which can lead to unnecessary patient doses. Inadequate initial training on CR technology explains in large proportion of the inappropriate use of exposure parameters.**

**PACS numbers: 87.50.up, 87.59bd**

**Key Words: X-rays, c**omputed radiography, entrance surface air kerma, low contrast objects, optimisation

INTRODUCTION

In the recent past, the use of film-screen imaging in countries with high resources has shifted to digital imaging. This transition is currently taking place in limited resource countries due to the increasing availability of low cost computed radiography (CR) systems on the market. This guarantees better access to modern and affordable imaging technology to wider population in this community than has been the case before. However, t**he transition to digital imaging in these countries needs adequate preparations before they are introduced to avoid potential increase in unnecessary in patient doses.** In Tanzania, replacement of film screen systems by CR imaging has increased from 3 in early 2000’s to 20 by the end of 2014 and the number is expected to increase by 3-folds by the end of year 2015.

The rapid increase is associated with intention of the Government to equip all public regional and district hospitals with such facilities during this year. The principal motivation behind the transition to CR imaging in the country is due to the expected reduction of the running costs of the dark room operations. Another reason is the ability to adjust contrast and brightness (post image processing) to suit the radiologists’ needs. Tele-radiology practice is also a motivation in few hospitals where there are no radiologists or for the purpose of getting the second opinion on the image interpretation. A major limitation during this transition to CR imaging in the country is the fact that there were no prior preparations on the familiarization to this modern technology. In some hospitals, the dark room services were stopped as soon as the CR systems were commissioned, which could not assure smooth transition. In addition, little or no adequate training was provided to radiographers, radiologists or other physicians on the expected difference in selecting exposure parameters as well as in image appearance, which could influence the decisions during the diagnosis.

Already efforts with varying degree of success have been made to reduce the patient dose without sacrificing image quality as a result of patient dose monitoring programmes ^(1, 2, 3)^. In particular, studies have demonstrated the usefulness of phantoms in assessing the level of optimisation of parameters for the systems in clinical use ^(4, 5, 6)^. Due to the rapid increasing of CR systems there is a need to extend these to these studies to all referral hospitals. The objective of this study was to evaluate radiation doses to patients during chest and abdomen CR examinations and to assess the related level of optimisation at referral hospitals.

MATERIALS AND METHODS

**X-ray facilities**

The study was performed during the year 2013 at five referral hospitals in Tanzania. The hospitals are Arusha Lutheran Medical Centre (ALMC), Bugando Medical Centre (BMC), Aga Khan Medical Centre (AMC), Mbeya Consultant Hospital (MCH) and Hubert Kairuki Memorial Hospital (HKMH). For simplicity reasons, ALMC, BMC, AMC, MCH and HKMH will be referred to as Arusha, Bugando, Aga Khan, Mbeya and Hubert Kairuki hospital respectively. Each of these hospitals possesses one CR system, which is used for general purpose X-ray examinations and interprets the radiographic images. All radiographs of the studied patients for which doses are reported were used for diagnosis. Table 1 shows the type of equipment and CR systems used at the hospitals.

**Table1. The equipment and CR systems studied at each hospital. All Philips equipment was manufactured Phillips Medical System, Hamburg, Germany).Fuji imaging system and image plate were manufactured by Fuji Film Company Limited, Japan.**

| Hospital | equipment model | CR model | |
| --- | --- | --- | --- |
|  |  | imaging system | imaging plate (IP) and IP cassette |
| Arusha | GE Proteus XR/a (General Electric, USA) | Philips FCR T2 Prima | ST-VI and IP cassette type CC |
| Bugando | Philips Duo diagnostic | Philips FCR Capsula | ST-VI and IP cassette type CC |
| Aga Khan | Philips Optima 50 Diagnos | Philips FCR Capsula CR-FR- 359 | ST-VI and IP cassette type CC |
| Mbeya | Philips Bucky Diagnost | Philips PCR Eleva S | ST-VI and IP cassette type CC |
| Hubert Kairuki | Toshiba, Varian (Toshiba, Japan) | Kodak Care stream classic CR system | Kodak, IP cassette type CC |

**ESAK Determination**

A methodology recommended in the International code of practice for dosimetry in diagnostic radiology ^(7)^ was applied to determine ESAK to patients undergoing chest *posteroanterior* (PA) and abdomen *anteroposterior* (AP) examinations. Important demographic and exposure data during the corresponding chest PA and abdomen AP examinations of 10 adult patients were collected for each projection at each facility. These included the age, gender, height, weight, tube potential, tube loading, focus skin distance (FSD) and the field size. The height and weight ranges were 164-173 cm and 65.6-70.5 kg respectively. All radiographs of the studied patients for which doses are reported were used for diagnosis. The output and half value layer (HVL) were measured using a detector model XR (serial number R12-0145 version 01) connected to model Magic–Max Universal (serial number G 13-0133 version 01). Both the detector and the measuring assembly were manufactured and calibrated in 2011 by IBA dosimetry GmbH in Germany. The output for each equipment was measured at 500 mm focus to detector distance (FDD) and 10 mAs at 60, 70, 80, 90 and 120 kVp settings at a time. For Philips Duo-diagnostic equipment that do not have 70, 80 and 117 kVp settings, 71, 81 and 117 kVp settings respectively were selected instead.

The air kerma (K(d) from the equipment at particular tube potential (kVp) and tube loading (mAs) at a distance (d) of 1 m from the source was calculated using equation (1) as:


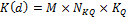
(1)

where *M* is the dosimeter reading at FDD, *N_KQ_* is calibration coefficient at reference beam quality *Q* (set to 1 during dosimeter calibration), *K_Q_* is the factor, which corrects for difference in the response of the dosimeter at the calibration quality *Q*, (stated to be ±5% in calibration certificate), *K_TP_* is the correction factor for temperature and pressure (which is 1 for semiconductor detector used). From *K(d)* measurements, the tube output, *Y(d)* in µGy per mAs was then calculated as the quotient of *K(d)* by *P_It_* as shown in equation (2):


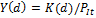
 (2)

where *K (d)* is the air kerma rate and *P_It_* is the tube loading during the exposure in mAs.

The incident air kerma (*K_i_*) is defined as the air kerma measured on the central beam axis at the position of the patient due to a beam but without the backscattered radiation. ESAK for each patent was assessed indirectly using *K_i_* values calculated using equation (3):


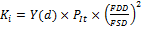
 (3)

where *Y(d)* is the output (mGy per mAs) of the tube at particular exposure settings*, P_It_* is the tube loading during the exposure of the patient and *FSD* is the distance measured from the tube focal spot to the skin entrance surface using a tape measure. ESAK was calculated by multiplying the incident air kerma with an appropriate backscatter factor (BSF) as in equation (4):


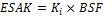
 (4)

It is known that the backscatter factor (BSF) is dependent on FSD, radiation field size (FS), tube filter, tube potential and half value layer (HVL). Therefore in this study the backscatter factors (BSFs) at 1000 mm FSD, 250 x250 mm FS, 2.5 mm Al filter and the measured half value layers (HVLs) were assumed ^(7)^.

.

# Optimisation of radiography technique

### *Tube potential*

### The assessment of the optimisation level of tube potential in clinical practice was studied by using Leeds TOR CDR 20 phantom (University of Leeds, United Kingdom). At each facility, the phantom was placed between two 30 x30 cm *p*[***olymethyl methacrylate***](http://www.google.co.tz/url?sa=t&rct=j&q=&esrc=s&source=web&cd=2&cad=rja&uact=8&sqi=2&ved=0CCIQFjAB&url=http%3A%2F%2Fen.wikipedia.org%2Fwiki%2FPoly%2528methyl_methacrylate%2529&ei=33qJVKSSG8nrUpu8gvgM&usg=AFQjCNEI5g2xM7VNTz16b1RPQN6BzeVNWg&bvm=bv.81456516,d.d24) (PMMA) plates each of 2 cm thick) to stimulate radiation scattering conditions and placed on chest stand. The tube was positioned as clinically used in chest PA set up i.e. directed towards the chest stand. The radiation field size was set up to 30 cm x 30 cm (to cover the phantom fully) and the exposure made at 60 kVp and 5 mAs. The selection of 5 mAs was based on the experience and the fact that the literature suggests that lower doses is more appropriate for digital detectors than for film-screen detectors ^(8)^. The imaging plate (IP) was read out and the same IP used for each subsequent exposures at 70/71, 80/81, 90 and 120/117 kVp settings as during output measurements. There was no fixed delay time allowed prior to IP reading to match this study with clinical conditions where usually such delays are in existent. The visibility of low contrast objects for the images obtained at different tube potential settings were assessed by three independent previously trained persons. Similar IP exposure procedure was undertaken for abdomen AP set up i.e. Leeds and PMMA phantom placed on top of patient table at the same tube potential tube loading and field size conditions. The level of optimisation of each projection was deduced at the tube potential that exhibited the maximum mean number of visible low contrast objects.

### *Tube loading*

In order to assess the optimisation level of tube load optimisation at each facility, the same phantom and procedure as during the assessment of the optimisation level of the clinical tube potential settings was used. The tube potential of 70 kVp was fixed while varying the tube loading settings at 1, 2.5, 5, 10, 20 and 40 mAs at a time for chest PA and abdomen AP projections. The selection of 70 kVp was based on the results of tube potential optimisation in previous subsection. The selection of 1-40 mAs range was based on the experience on the need to use low tube load values and the observed clinical practice in the studied hospitals, which covered up to 50 mAs. The level of optimisation of each projection was deduced at the tube loading value with the maximum mean number of visible low contrast objects.

RESULTS

The mean ESAK values for patients chest PA examinations are presented in Table 2. The ESAK values ranged from 0.3 to 0.37 mGy at all five facilities with intra facility ESAK variations varying from 1.4 to 7.7. The patient doses are largely similar to the international diagnostic reference level (IDRL) of 0.3 mGy ^(9)^ except at Aga Khan and Hubert Kairuki hospitals. Higher tube loading values in use at these hospitals provide a main explanation. Table 3 shows the mean ESAK values to patient during abdomen AP examinations. The ESAK values varied from 2 to 6 mGy with moderate intra hospital variations of 1.2 to 2.3. As for chest PA case, the ESAK values are similar to IDRL of 5 mGy ^(9)^ except at Mbeya hospital. Higher mean ESAK value at the latter hospital is mainly attributed to the slightly higher tube potentials in use.

**Table 2. Mean ESAK of adult patient during chest PA examinations at studied hospitals. The ratio of maximum to minimum ESAK is indicated as Max/Min.** The confidence intervals refer to one standard deviation

| Hospital | Mean height (mm) | Mean weight (kg) | Mean tube potential (kVp) | Mean tube loading (mAs) | ESAK | |
| --- | --- | --- | --- | --- | --- | --- |
|  |  |  |  |  | Mean mGy) | Max/Min |
| Arusha | 1670.7±27.6 | 69±2.7 | 72.7±1.3 | 10±0.20 | 0.31±0.1 | 1.4 |
| Bugando | 1688± 27.8 | 70±2.4 | 60.9±0.6 | 6.6 ± 0.1 | 0.16±0.01 | 1.9 |
| Aga Khan | 1651.4±23.0 | 66±1.3 | 67.1±0.4 | 19.2±0.6 | 0.34±0.01 | 1.8 |
| Mbeya | 1688.4±26.8 | 70.3±2.7 | 60.2±0.5 | 9.9±0.5 | 0.25±0.04 | 7.7 |
| Hubert Kairuki | 1713.9±23.9 | 70.5±2.3 | 71.5±1.4 | 20.85±0.8 | 0.37±0.03 | 1.9 |

**Table 1**. Mean ESAK of adult patient during abdomen AP examinations at studied hospitals. The ratio of maximum to minimum ESAK is indicated as Max/Min. The confidence intervals refer to one standard deviation

| Hospital | Mean height (mm) | Mean weight (kg) | Mean tube potential (kVp) | Mean tube loading (mAs) | ESAK | |
| --- | --- | --- | --- | --- | --- | --- |
|  |  |  |  |  | Mean (mGy) | Max/Min |
| Arusha | 1644±22.6 | 72.2±2.3 | 78.70±0.2 | 50±0.2 | 3.1±0.1 | 1.2 |
| Bugando | 1679±32.9 | 67.8±2.2 | 77.8±0.4 | 29.3±1.6 | 2±0.1 | 1.6 |
| Aga Khan | 1659±23 | 70.3±2.4 | 78.2±2.3 | 28.1±3.1 | 2.4±0.02 | 3.3 |
| Mbeya | 1730±29.4 | 65.58±2.2 | 100±0.3 | 49.9±1.7 | 6±0.03 | 1.4 |
| Hubert Kairuki | 1694±31.3 | 69.2±2.7 | 83.6±2.9 | 55.5±0.2 | 4±0.3 | 2.3 |

Fig. 1 shows the results of mean visibility score of the low contrast objects of the phantom studied at different tube potentials. As expected, the visibility decreased with increasing tube potential since CR detectors are more efficient at low than at high tube potentials ^(8)^. The exception was observed at 90 and 120 kVp for Arusha hospital. The exception is likely to be attributed to equipment (machine and laser scanner) characteristics. The results indicate that the visibility beyond 110 kVp was less than 50% suggesting that the region is of limited optimisation.

The variation of the mean visibility score of the phantom studied as a function of different tube load settings is presented in Fig. 2. It can be seen that generally, the visibility beyond 2.5 mAs was roughly decreasing with increasing tube loading. This was expected taking into account that CR detectors are most sensitive at low than high dose values ^(8)^. The increasing trend below 2.5 mAs (Fig. 2) can be explained by the higher phantom attenuation over this tube loading region. The results show that the visibility was nearly constant beyond 20 mAs suggesting that higher tube load values would not be useful in details visibilities. This implies that the use of such values would potentially increase unnecessary dose to patients.


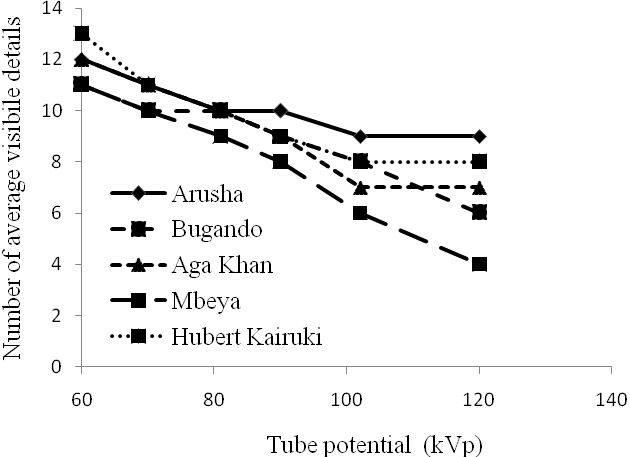


Fig. 1. The variation of the mean number of visible details as a function of tube potential


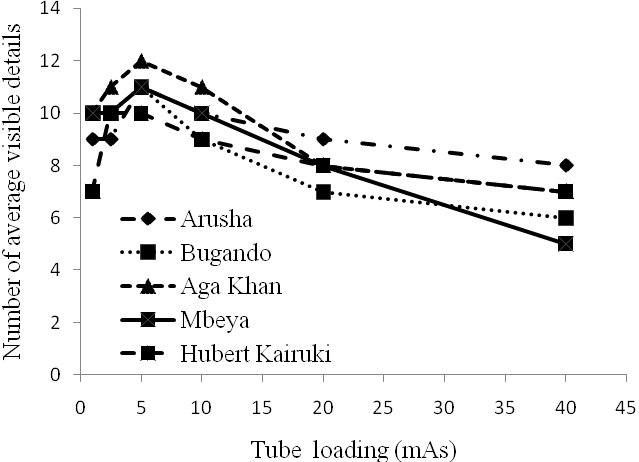


Fig. 2. The mean number of visible details as a function of tube loading

DISCUSSION

**X-ray technique and patient dose**

The survey of X-ray techniques in clinical use and the related patient doses in diagnostic radiology and is important to identify non optimised practices. A number of studies have confirmed the usefulness of such studies and the related comparisons with good practices as a key step towards achieving optimised clinical practices ^(4, 5. 10)^. The results from this study have shown that relatively low tube potential values are used for chest PA at all studied X-ray facilities (Table 1) in comparisons to usual high kVp technique in film-screen systems. This is important since digital detectors are known to have high absorption at low kVp, which enhance visualisation of details at such low X-ray energies ^(4, 5)^. The use of such low kVp values is also demonstrated for abdomen AP projections at majority X-ray facilities (Table 2). This study has also demonstrated that majority patient doses during these examinations are largely below the recently published IDRLs with few exceptions (Tables 1 and 2).

**Comparisons with other studies**

The results of patient dose varied from **0.16 to 0.37 mGy for chest PA and from 2 to 6 mGy for abdomen AP. Previous study in the country showed that ESAK values during chest CR ranged from 259-367 µGy implying that the present results values are relatively higher ^(6)^. This suggests the need to continuously monitor the patient doses. In a study conducted by Veldkamp** *et al.*  **^(10)^, mean entrance surface dose for average adult chest CR was 109 µGy, implying lower value than the results in the present study. Table 4 summarizes the comparison of the results in the present study with the corresponding values in film screen systems in countries with typical economical status as that under which the present study was conducted ^(11)^. It can be seen that comparable results were achieved for chest PA while mixed results observed for abdomen AP X-ray examinations.**

Table 4. Comparisons of patient CR doses in this study with patient doses in film screen systems. All patient doses are in mGy units ^(11)^.

| X-ray projection | Present study | Madagascar | Sudan | Ghana | Tanzania |
| --- | --- | --- | --- | --- | --- |
| Chest PA | 0.16-0.37 | 0.29 | 0.21 | 0.1 | 0.3 |
| Abdomen AP | 2-6 | 3.92 | 1.5 | 10.3 | 0.9 |

**Optimisation of X-ray techniques**

The results of optimisation (Fig. 1 and Fig. 2) bear some limitations and therefore require appropriate interpretations. First, the Leeds test object employed during the optimisation was designed to be used at 70 kVp with 1 mm Al filtration mainly to test the performance of the IP detector. In this study, this test object was placed between two PMMA phantoms each of 2 mm thickness to simulate the scatter conditions under typical patient’s X-ray examination. Second, the phantom thickness (Leeds test object and 4 mm thick PMMA) may not represent the scatter radiation in real situations where thicker patient sizes are common (Tables 1 and 2). However, assuming proportionality between the study conditions and the real situation, the results show that the optimal tube potential is likely to be below 90 kVp (Fig. 1), which closely resemble the tube potentials in use at majority studied X-ray facilities. However, under the same assumptions, the results suggest that the optimal tube loading to be around 5 mAs (Fig. 2). The results suggest that the use of tube load values beyond 40 mAs would bear no benefit on details visualisation but would potentially contribute to unnecessary dose to patients. Continued training of radiology personnel the use of appropriate X-ray parameters in CR technology is therefore of utmost importance.

CONCLUSION

**Patient dose and optimisation levels during chest and abdomen CR examinations have been evaluated at five facilities. The study has demonstrated the mean ESAK values to be comparable to other published results. Assuming similar patient and phantom attenuation, the optimisation at four facilities was consistent with phantom evaluations for tube potential settings in use. However, all facilities seemed to use higher tube load values suggesting that unnecessary doses can be imparted to patients. Inadequate initial training on CR technology forms a main explanation for such practice and the related improvement offers a sustainable solution.** The results should be useful to serve as a good experience for early intervention where non optimal X–ray examinations are practiced.

FUNDING

The work was supported by University of Dar es Salaam as a part of his Master of Science in Physics degree study.

ACKNOWLEDGEMENT

The authors are indebted to the Managements of Arusha Lutheran Medical Centre, Bugando Medical Centre, Aga Khan Medical centre, Mbeya Consultant Hospital and Hubert Kairuki Memorial Hospital for their permission to use the hospital facilities and the provision of related logistics. The authors would like also to thank the International Atomic Energy Agency to provide the dosimetry equipment under RAF9044 project that was used during this study. They also thank the staff members of Physics Department, University of Dar es Salaam for their constructive comments.

1. Weatherburn, G.C., Bryan, S., Davies, J.G. Comparison of doses for bedside examinations of the chest with conventional screen-film and computed radiography: Results of a randomized controlled trial. Radiology. 2000; 217:707-712.
2. Bacher, K., Smeets, P., Bonnaresns, K., De Hauwere, A., Verstaete, K., Thierens, H. Dose reduction in patients undergoing chest imaging: Digital amorphous silicon flat panel detector radiography versus conventional film-screen radiography and phosphor –based computed radiography. Am. J. Roentgenol. 2003; 181: 923-929.
3. Aldrich, J.E., Duran, E., Dunlop, P., Mayo, J.R., Optimization of dose and image quality for computed radiography and digital radiography. J. Digit. Imag. 2006; 19(2): 126-133
4. McEntee, M., Frawley, H., Brenna, P.C. A comparison of low contrast performance for amorphous Silcon/Caesium iodide direct radiography with a computed radiography: A contrast detail phantom study, Radiography:(2007);13 (2): 89-94.
5. Sandborg, M, Tingberg, A, Ullman, G, Dance, D.R., Carlsson, G.A. (2006). Comparison of clinical and physical measures of image quality in chest and pelvis computed radiography at different tube voltage. Med Phys. 2006; 33 (11): 4169-4175.
6. Muhogora, W.E., Trianni, A., Toso, F., Devetti, A., Padovani, R, Msaki., P., Kazema, R. Comparison of image quality at patient dose for chest x-ray examinations on conventional and low cost computed radiography systems. Radiography 2012; 18: 275-278.
7. International Atomic Energy Agency. Dosimetry in diagnostic radiology: An International Code of Practice, Technical Reports Series No. 457, IAEA, Vienna 2007.
8. American Association of Physicists in Medicine (AAPM). Acceptance testing and quality control of photostimulable phosphor imaging systems, AAPM Report No. 95, Report of AAPM Task group 10, AAPM, 2006.
9. European Commission. Guidance on diagnostic reference levels (DRLs) for medical exposures. Radiation protection 109, EC, 1999.
10. Veldkamp, W.H., Kroft, L.J.M., Boot, M.V., Mertens, B.J.A., Gelejeins, J. Contrast-detail evaluatin and dose assessment of eight digital chest radiography systems in clinical practice. Eur. Radiol. 2006; (16): 333-341.
11. Muhogora, W. E., Ahmed, N.A., Almosabihi, A., Alsuwaidi, J.S., Beganovic, A., Ciaj-Bjelac, O., Kabuya, F.M., Krisanachinda, A., Milakovic M., et al. Patient doses in radiographic examinations in 12 countries in Asia, Africa and Eastern Europe: Initial results from IAEA projects, Am. J. Roentgenol. 2008;190 (6):1453-1461.
