# Supplementary material for: Assessment of patient dose and optimization levels in chest and abdomen CR examinations at referral hospitals in Tanzania
Source: J Appl Clin Med Phys. 2015 Sep 8;16(5):435–41. doi: 10.1120/jacmp.v16i5.5614 (PMC5690155; doi:10.1120/jacmp.v16i5.5614)
Supplement: Supplementary file 2 — Supplementary Material [file ACM2-16-435-s002.doc]

**Abstract: The aim of this study was to evaluate the level of patient doses in chest and abdomen CR examinations, and that of optimisation at five facilities. The international code of practice for dosimetry in diagnostic radiology was applied to determine the entrance surface air kerma (ESAK) to patients. The level of optimisation was assessed from low contrast objects scores of phantom images at different exposures. The results show that mean ESAK varied from 0.16 to 0.37 mGy for chest PA and from 2 to 6 mGy for abdomen AP. Assuming similar patient and phantom attenuations, the optimisation performed at four facilities was consistent with phantom evaluations in terms of tube potential settings in use. However, all facilities seemed to operate at higher tube load values, which can lead to unnecessary patient doses. Inadequate initial training on CR technology explains in large proportion of the inappropriate use of exposure parameters.**

**The following change has been take place in this abstract as I requested to make Abstract to reflect objectives.**

**Line 1: sentence rewritten as “The aim of this study was to evaluate the radiation doses to patients during chest and abdomen CR examinations, and** assess the related level of optimisation **at five referral hospitals in Tanzania”.**

**Line 7: sentence rewritten as “However, all facilities seemed to operate at higher tube load values above 5mAs for chest examination, which can lead to unnecessary patient doses”**
